# Quantitative Trait Loci and Transcriptome Analysis Reveal Genetic Basis of Fiber Quality Traits in CCRI70 RIL Population of *Gossypium hirsutum*

**DOI:** 10.3389/fpls.2021.753755

**Published:** 2021-12-16

**Authors:** Xiao Jiang, Juwu Gong, Jianhong Zhang, Zhen Zhang, Yuzhen Shi, Junwen Li, Aiying Liu, Wankui Gong, Qun Ge, Xiaoying Deng, Senmiao Fan, Haodong Chen, Zhengcheng Kuang, Jingtao Pan, Jincan Che, Shuya Zhang, Tingting Jia, Renhui Wei, Quanjia Chen, Shoujun Wei, Haihong Shang, Youlu Yuan

**Affiliations:** ^1^State Key Laboratory of Cotton Biology, Institute of Cotton Research, Chinese Academy of Agricultural Sciences, Anyang, China; ^2^College of Agriculture, Engineering Research Centre of Cotton of Ministry of Education, Xinjiang Agricultural University, Ürümqi, China; ^3^Institute of Cotton, Hebei Academy of Agriculture and Forestry Sciences, Shijiazhuang, China; ^4^Cotton Sciences Research Institute of Hunan, National Hybrid Cotton Research Promotion Center, Changde, China; ^5^School of Agricultural Sciences, Zhengzhou University, Zhengzhou, China

**Keywords:** *Gossypium hirsutum*, RIL, fiber quality, quantitative trait loci, RNA-seq

## Abstract

Upland cotton (*Gossypium hirsutum*) is widely planted around the world for its natural fiber, and producing high-quality fiber is essential for the textile industry. CCRI70 is a hybrid cotton plant harboring superior yield and fiber quality, whose recombinant inbred line (RIL) population was developed from two upland cotton varieties (sGK156 and 901-001) and were used here to investigate the source of high-quality related alleles. Based on the material of the whole population, a high-density genetic map was constructed using specific locus-amplified fragment sequencing (SLAF-seq). It contained 24,425 single nucleotide polymorphism (SNP) markers, spanning a distance of 4,850.47 centimorgans (cM) over 26 chromosomes with an average marker interval of 0.20 cM. In evaluating three fiber quality traits in nine environments to detect multiple environments stable quantitative trait loci (QTLs), we found 289 QTLs, of which 36 of them were stable QTLs and 18 were novel. Based on the transcriptome analysis for two parents and two RILs, 24,941 unique differentially expressed genes (DEGs) were identified, 473 of which were promising genes. For the fiber strength (FS) QTLs, 320 DEGs were identified, suggesting that pectin synthesis, phenylpropanoid biosynthesis, and plant hormone signaling pathways could influence FS, and several transcription factors may regulate fiber development, such as *GAE6*, *C4H*, *OMT1*, *AFR18*, *EIN3*, *bZIP44*, and *GAI*. Notably, the marker D13_56413025 in *qFS-chr18-4* provides a potential basis for enhancing fiber quality of upland cotton *via* marker-assisted breeding and gene cloning of important fiber quality traits.

## Introduction

Upland cotton (*Gossypium hirsutum* L., 2*n* = 52) is a widely planted cash crop providing natural fiber. Due to its excellent environmental adaptability and yield, among the four cultivated tetraploid species, it is *G. hirsutum* that contributes almost 95% of the harvested cotton production (Yoo and Wendel, 2014). Given the increasing demand for high-quality fiber from the textile industry and the role of multigenes’ contribution and environmental factors, it is the goal of cotton breeders worldwide to develop new upland cotton varieties simultaneously featuring superior fiber quality and high yield.

Genome sequencing studies of *G. raimondii* (Paterson et al., 2012; Wang K. et al., 2012), *G. arboreum* (Li et al., 2014; Du et al., 2018; Huang et al., 2020), *G. barbadense* (Liu X. et al., 2015; Yuan et al., 2015; Hu et al., 2019), and *G. hirsutum* (Li et al., 2015; Zhang T. et al., 2015; Hu et al., 2019; Wang M. et al., 2019; Huang et al., 2020) using next-generation and third generation sequencing technology have provided a solid basis for constructing the genetic map of cotton and understanding further its functional genomics. Benefiting from rapid progress in sequencing and DNA marker technologies, marker-assisted breeding has become one of the most efficient tools to help breeders globally improve agronomic traits and shorten the breeding cycle in multiple key crops. Recently, specific locus-amplified fragment sequencing (SLAF-seq) and genotyping-by-sequencing have merged as efficient tools largely applied to upland cotton for exploring its genotypic variants. SLAF-seq has several distinguishing advantages: (i) deep sequencing to ensure genotyping accuracy; (ii) reduced representation strategy to reduce sequencing costs; and (iii) a double barcode system for large populations (Sun et al., 2013). Many cotton genetic linkage maps have been constructed and quantitative trait loci (QTL) identified using different kinds of populations and DNA markers (Ulloa et al., 2002, 2005; Rong et al., 2004; Chen et al., 2009; Sun et al., 2012; Zhang et al., 2012, 2016; Zhou et al., 2017; Zhang et al., 2020; Hulse-Kemp et al., 2015; Shi et al., 2015; Wang H. et al., 2015; Wang Y. et al., 2015; Yuan et al., 2015; Zhang Z. et al., 2015; Li et al., 2016; Liu et al., 2017; Qi et al., 2017; Chandnani et al., 2018; Diouf et al., 2018; Zou et al., 2018; Wang et al., 2021). Since the fiber quality is a quantitative trait and one controlled by multiple genes, QTLs might cumulatively contribute to its phenotypic variation, which provides a reasonable way to improve fiber quality *via* marker-assisted selection (MAS) (Paterson et al., 2003; Rong et al., 2004; Zhang et al., 2012; Wang H. et al., 2015; Wang Y. et al., 2015).

Based on an assessment of previous research, fiber development could be classified into four stages: initiation [−3 to 3 days postanthesis (DPA)], elongation (3–23 DPA), secondary wall biosynthesis (20–40 DPA), and maturity (40–50 DPA) (Basra and Malik, 1984; Kim and Triplett, 2001; Lee et al., 2006, 2007; Haigler et al., 2012; Mathangadeera et al., 2020; Patel et al., 2020). Initiation, elongation, and secondary wall biosynthesis stages will determine fiber quality traits, namely fiber length (FL), fiber strength (FS), and fiber micronaire (FM), respectively. Transcriptome sequencing, better known as RNA-Seq, which takes full advantage of gene expression and transcriptional regulation, has proven itself a robust and suitable procedure for analyzing the transcriptome profile during various stages of fiber development (Applequist et al., 2001; Gilbert et al., 2014; Yoo and Wendel, 2014; Islam et al., 2016; Li P.-t. et al., 2017; Li X. et al., 2017; Lu et al., 2017; Zou et al., 2019; Jiang et al., 2021).

The hybrid variety “CCRI70”, a nationally approved variety in China released in 2008, is capable of good yield and has high fiber quality, whose recombinant inbred line (RIL) population consisting of 250 individuals was developed to investigate the source of high-quality related alleles for further upland cotton breeding (Zou et al., 2018; Deng et al., 2019). In this study, the CCRI70 F_8:9_ RIL population was used to evaluate fiber quality performance in nine environments and to construct a genetic linkage map. The whole-genome-based high-density genetic map contained 24,425 single nucleotide polymorphism (SNP) markers spanning a distance of 4,850.47 cM. Meanwhile, 289 QTLs for the three fiber quality traits and seven QTL clusters were identified. Accompanying the RNA-Seq analysis done for the whole process of fiber development, differentially expressed genes (DEGs) in those obtained QTLs and clusters were also identified, which provides new and timely insights into the genetic basis of fiber development and fiber quality traits.

## Materials and Methods

### Plant Materials

The 250 individual F_8:9_ RILs were developed from the hybrid cotton variety CCRI70 whose parents are “sGK156” (P1) and “901-001” (P2); this population was developed from 2011 onward at the experimental farm of the Institute of Cotton Research, Chinese Academy of Agricultural Sciences (CAAS), in Anyang, Henan Province. The CCRI70 (F_1_) was obtained and planted in Sanya, where the F_2_ seeds were harvested. Shuttle breeding was applied to further develop the population during 2012–2016 between Anyang and Sanya. Full details of the procedure used to generate the RIL populations were reported already (Zou et al., 2018).

In 2017, the RIL population was planted in double-row plots 5-m long, 80 cm apart, and with a 25-cm spacing between adjacent plants. “Lumianyan28” was also planted, as a control, for every 19 RILs at the Anyang experimental station of Institute of Cotton Research of CAAS, in Henan Province. Leaf samples of the RIL population were collected for sequencing. The P1and P2, and the two RILs, MBZ70-053 (L1) and MBZ70-236 (L2), known to differ in their fiber quality performance were designated for RNA sequencing. Concerning the above four types of plant materials, P2 and L1 performed high-fiber quality, and P1 and L2 showed low-fiber quality in terms of their FL and FS. The day of anthesis was marked as 0 DPA, and flowers were marked by hanging labels with the flowering date recorded. Cotton bolls were collected in the morning at 5, 10, 15, 20, 25, and 30 DPA with three biological replicates, with at least five bolls collected in each replicate. The fiber samples were collected using sterilized medical scalpel and tweezers. Then all the samples were frozen in liquid nitrogen and stored at −80^°^C for RNA-Seq analysis.

### Evaluation in Multiple Environments and Phenotypic Data Analysis

The 250 CCRI70 RILs and the two parents were planted in at least two replications in nine environments across 2 years and six locations, including the F_5:7_ and F_5:8_ families, reported previously (Zou et al., 2018). In 2015, the RIL population was planted in Anyang of Henan Province (15AY, 36°10′N, 114°35′E), Linqing of Shandong Province (15LQ, 36°68′N, 115°72′E), and Alaer of the Xinjiang Autonomous Region (15ALE, 40°22′N, 80°30′E). In 2016, the population was planted in Anyang (16AY), Linqing (16LQ), Alaer (16ALE), Changde of Hunan Province (16CD, 29°2′N, 111°41′E), Shihezi (16SHZ, 44°27′N, 85°94′E), and Kuerle (16KEL, 41°68′N, 86°06′E) of the Xinjiang Autonomous Region.

Thirty mature, fully opened bolls from every plot were harvested to test their fiber quality (i.e., FL, FS, and FM traits) by using an HVI1000 (Uster Technologies, Switzerland) with the HVICC Calibration at the Cotton Quality Supervision, Inspection, and Testing Center, Ministry of Agriculture, Anyang, China (Zhang Z. et al., 2015). The descriptive statistics of these fiber quality traits were calculated using SPSS 20.0 and Microsoft Excel 2010 software. IciMapping 4.1 was used to analyze the traits’ heritability (Li et al., 2007; Meng et al., 2015).

### Recombinant Inbred Line Population Library Construction and Sequencing

Leaf samples of the RIL population were used to extract genomic DNAs; this is done using the TaKaRa MiniBEST Plant Genomic DNA Extraction kit (TaKaRa, Dalian). According to the pilot experiment and the preexperiment *in silico* simulation, SLAF libraries for 250 RILs were constructed by using two endonucleases in combination, *Hae*III and *Ssp*I (New England Biolabs, NEB, United States), to digest the genomic DNA (Zhang et al., 2017). The details and procedures of the SLAF-seq strategy are described in Zhang J. et al. (2015). Paired-end (PE) sequencing libraries of the parents with insert sizes ranging from 200 to 500 bp were generated according to the manufacturer’s instructions (Illumina, San Diego, CA, United States). Likewise, following the manufacturer’s recommendations, all PE sequencing (each 125 bp fragment of the 250 RILs and each 150 bp fragment of parents) were conducted on the Illumina Hi-Seq 2500 system (Illumina, San Diego, CA, United States).

### Genotyping, Analysis of Single Nucleotide Polymorphism Markers, and Linkage Map Construction

The identification and genotyping of SNP markers were implemented using the procedures of Sun et al. (2013) and Zhang J. et al. (2015). After removing the adapters and filtering out any low quality reads (i.e., quality score < 20e), the reads belonging to each RIL were recognized by their unique duplex barcode sequences (Zhang Z. et al., 2015). The clean data set of the population was then mapped onto the *G. hirsutum* reference genome (Hu et al., 2019) using BWA software (Li and Durbin, 2009). Reads mapping onto the same position with a more than 95% shared identity were recorded as a single locus. GATK (McKenna et al., 2010) was used to process the bam files, and these were then filtered according to GATK’s recommended parameters. The SNP markers were genotyped under the sequencing depth of parents with more than 10-fold and individuals above 1-fold. Before constructing the genetic linkage map, SNP markers underwent further filtering by removing those markers with no position or at least two positions; those which showed no polymorphism between parents; those that were heterozygous in either parent; and those with a missing rate above 50%, or with segregation distortion having a *p*-value < 0.001 for the chi-square test (Zhang Z. et al., 2015).

After genotyping and filtering the SNP marker, the genetic linkage map was built using MSTmap software and referring to the reference genome (Hu et al., 2019). To be considered for linkage map construction, markers had to have a LOD (log of odds) threshold between 4 and 20 (Wu et al., 2008). Next, SNP markers in the linkage group from the same chromosome were merged together, and these SNP markers per chromosome were then regulated again by MSTmap.

### Quantitative Trait Loci and Quantitative Trait Loci Cluster Identification

The QTLs for the FS, FL, and FM traits in the nine environments were identified using WinQTLCart 2.5 software (Wang et al., 2007) and applying the composite interval mapping (CIM) method (Zeng, 1994). LOD thresholds were determined with 1,000 permutations at a 1-cM walk (*p* = 0.05) (Churchill and Doerge, 1994), and QTLs designated so that their LOD scores were above the threshold. The additive QTL was named as “q” with the trait name, followed by the corresponding chromosome number and QTL number according to the genetic position. The QTLs that could be detected in no less than three environments were recognized as being stable QTLs (Sun et al., 2012). Places on the genetic map where confidence intervals overlapped stable QTLs for different traits were considered to be QTL clusters (Zhang et al., 2020), in which genome fragments affect more than one trait. Based on the physical position of markers for stable QTLs and QTL clusters, according to their annotation (Hu et al., 2019), those genes located internally were deemed “promising genes” (except the QTLs longer than 10 MB). QTL clusters were visualized using MapChart 2.2.

To compare our stable QTLs with those found in previous studies, the CottonQTLdb database^1^ was searched for using the physical positions of the border markers of QTLs (Rong et al., 2007; Lacape et al., 2010; Said et al., 2013, 2015; Fang et al., 2014). As we used the newly published reference genome, we mapped our QTL results on the former (Zhang T. et al., 2015) using Bowtie software (Langmead et al., 2009). Locating the physical confidence intervals of previous QTLs based on simple sequence repeat (SSR) markers using CottonFGD database,^2^ *via* comparison with the CottonQTLdb database, the former study of CCRI70, the SNP loci of genome-wide association studies (GWASs) (Fang et al., 2017a,b,c; Huang et al., 2017; Ma et al., 2018), and the QTLs with overlapping confidence intervals on the physical map were considered as the same QTL.

### Transcriptome Sequencing and RNA-Seq Analysis

Total RNA from each sample was extracted following the manufacturer’s protocol with the RNAprep Pure Plant Kit (Polysaccharides & Polyphenolics-rich, Tiangen, Beijing, China). The quantification and purity of the RNA were respectively assessed by a NanoDrop 2000 spectrophotometer (Thermo Fisher Scientific, Waltham, MA, United States) and a NanoPhotometer spectrophotometer (IMPLEN, CA, United States). The RNA integrity was confirmed using the RNA Nano 6000 Assay Kit of the Bioanalyzer 2100 system (Agilent Technologies, CA, United States). Next, according to the manufacturer’s recommendations, 2 μg RNA per sample was used for the transcriptome library construction, implemented with the Illumina TruSeq™ RNA Sample Preparation Kit (Illumina, San Diego, CA, United States). In total, 72 libraries were separately sequenced using Illumina Novaseq 6000 (BerryGenomics Co., Ltd., Beijing, China) for the two RILs and the two parents at six developing stages (5, 10, 15, 20, 25, and 30 DPA) with three biological replicates (4 × 6 × 3 = 72).

Trimmomatic software was then utilized to process all the generated raw data in the Fastq format (Bolger et al., 2014). After removing those reads having the adapter, poly-N (*N* ≥ 10%), and low-quality reads (more than half of the bases with a Phred quality ≤ 3), the clean data set was finally generated. Meanwhile, the GC percentage and Q30 were calculated to evaluate the quality of the clean data. HISAT2 v2.1.0 (Pertea et al., 2016) under its default parameters was used to carry out the sequence alignments. The fragments per kilobase of exon per million reads (FPKM) values of genes were quantified by StringTie v1.3.5 (Pertea et al., 2015) and these were then subjected to a Pearson correlation analysis to reveal their correlation coefficients. Those samples with a correlation coefficient <0.8 between the biological replicates were removed from the dataset. Bcftools 1.8 and snpEff 5.0, each set to its recommended parameters, were used to respectively identify and annotate the SNPs in the RNA-Seq data (Cingolani et al., 2012).

### Analysis of Differentially Expressed Genes and Annotation of Promising Genes

Based on the gene count number of each sample, the DESeq2 package for R was run to identify the DEGs (Love et al., 2014), for which the dual screening criteria were an FDR value < 0.05, and | log_2_FoldChange| > 1 between each pairwise comparison. The DEGs were obtained from vertical comparisons in the superior trait group (i.e., high fiber quality group, L1 and P2) *vis-à-vis* the inferior trait group (i.e., low fiber quality group, L2 and P1) at six stages, and also from horizontal comparisons within each group between the different developmental stages.

To predict the gene function and related pathways of the promising genes and DEGs detected during the course of fiber development, the BLASTX program, GO databases, and KAAS^3^ based on Kyoto Encyclopedia of Genes and Genomes (KEGG) were all employed (Altschul et al., 1990; Wu et al., 2006; Moriya et al., 2007; Xie et al., 2011). To identify the transcription factors of the promising genes, the PlantTFDB^4^ resource was queried (Jin et al., 2014, 2015, 2016; Tian et al., 2020).

### Weighted Gene Co-expression Network Analysis and Hub Genes Identification

A weighted gene co-expression network analysis (WGCNA) was performed and WGCNA R package was used to identify modules and hub (or highly correlated) genes that were strongly associated with the fiber development stages (Langfelder and Horvath, 2008). Those DEGs, with a coefficient (>0.6) within each sample, were subsequently clustered into different modules (Pei et al., 2017). The hub genes were selected according to the module membership (KME) values.

## Results

### Phenotypic Data Analyses of Fiber Quality Traits

**FIGURE 1:**
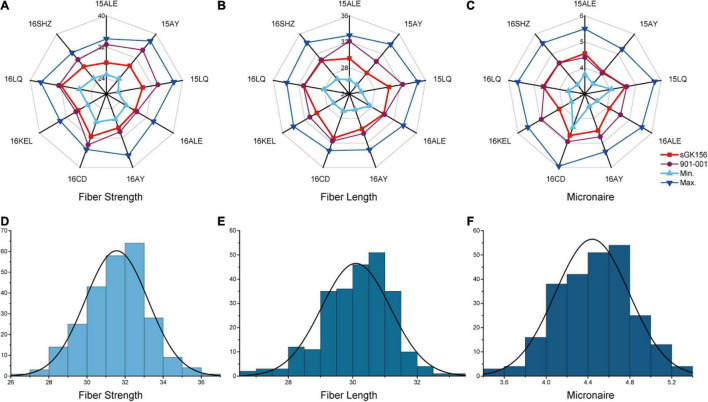
The phenotypic data analysis of the parents and two RILs in nine environments. The phenotypic data analysis of the parents and two RILs for **(A)** fiber strength, **(B)** fiber length, and **(C)** micronaire. Normal distribution analysis of **(D)** fiber strength, **(E)** fiber length, and **(F)** micronaire.

The phenotypic data for the CCRI70 RIL population were selected and evaluated in nine environments during 2015 and 2016 (Figures 1A–C). The range in the difference between the two parents in nine environments for the FS, FL, and FM traits was 0.55–5.25 cN/Tex, 0.05–2.65 mm, and 0–0.8 units, respectively (Supplementary Table 1), with significant differences found for FS and FL. In addition, all three traits for fiber quality had approximately normal distributions whose absolute skewness values did not exceed 0.5, and they were characterized by transgressive segregation with respect to parental performance in all nine environments (Figures 1D–F). L1 and L2 respectively had excellent and poor performance in terms of fiber quality in all nine testing environments (Supplementary Table 1). When calculated and compared across the nine environments, the 15LQ site had the highest heritability (no less than 75% for FS, FL, and FM), whereas 16ALE had the lowest (no more than 60% for FS, FL, and FM), a disparity perhaps arising from environmental factors. Apart from 16ALE, in the eight environments, the heritability for FS, FL, and FM traits were 60.8–80.1%, 65.29–77.6%, and 66.0–78.2%, respectively (Supplementary Table 1). The two-way analysis of variance (ANOVA) revealed highly significant effects of genotype (G), the environment (E), and the interaction between genotype and the environment (G × E) on all three traits. Importantly, genotype explained the most variance while the G × E interaction was responsible for more than 25% of the variation in each of the three traits (Table 1).

**TABLE 1:** ANOVA for fiber quality traits across multiple environments.

Trait	Source	Df	Sum_Sq	Mean_Sq	F_value	Percentage (%)	Pr (>F)
FS	Genotype (G)	249	8690	34.9	18.615	47.24	<2e-16***
	Environment (E)	8	4955	619.3	330.336	26.94	<2e-16***
	G × E interaction	1992	4750	2.4	1.272	25.82	1.63e-08***
FL	Genotype (G)	249	3926	15.77	20.219	59.85	<2e-16***
	Environment (E)	8	737	92.14	118.144	11.23	<2e-16***
	G × E interaction	1992	1897	0.95	1.221	28.92	2.26e-06***
FM	Genotype (G)	249	313.9	1.261	19.291	42.67	<2e-16***
	Environment (E)	8	235.6	29.446	450.539	32.03	<2e-16***
	G × E interaction	1992	186.1	0.093	1.429	25.30	<2e-16***

***, significant at P ≤ 0.001.

### Analysis of Sequencing Data and Single Nucleotide Polymorphism Markers

After library construction, sequencing, and filtering, 1.1 TB of clean data was obtained, for which the Q30 bases were 94.76% and its mapping ratio was 99.51%. Specifically, 82.01 GB per each parent and 3.72 GB per each RIL were obtained; comparing both to the upland cotton reference genome (2.14 GB), indicating that the sequencing depth of our study was 35.41-fold for each parent and 1.61-fold per progeny, respectively (Supplementary Table 2).

A total of 1,943,288 SNPs were detected in sGK-156 whereas 2,073,924 SNPs were identified in 901-001, whose homozygosity SNP markers amounted to 8,11,619 and 8,82,936, respectively. After filtering these markers, 24,425 SNP markers were retained for the genetic map’s construction.

### Construction of the Genetic Map

**FIGURE 2:**
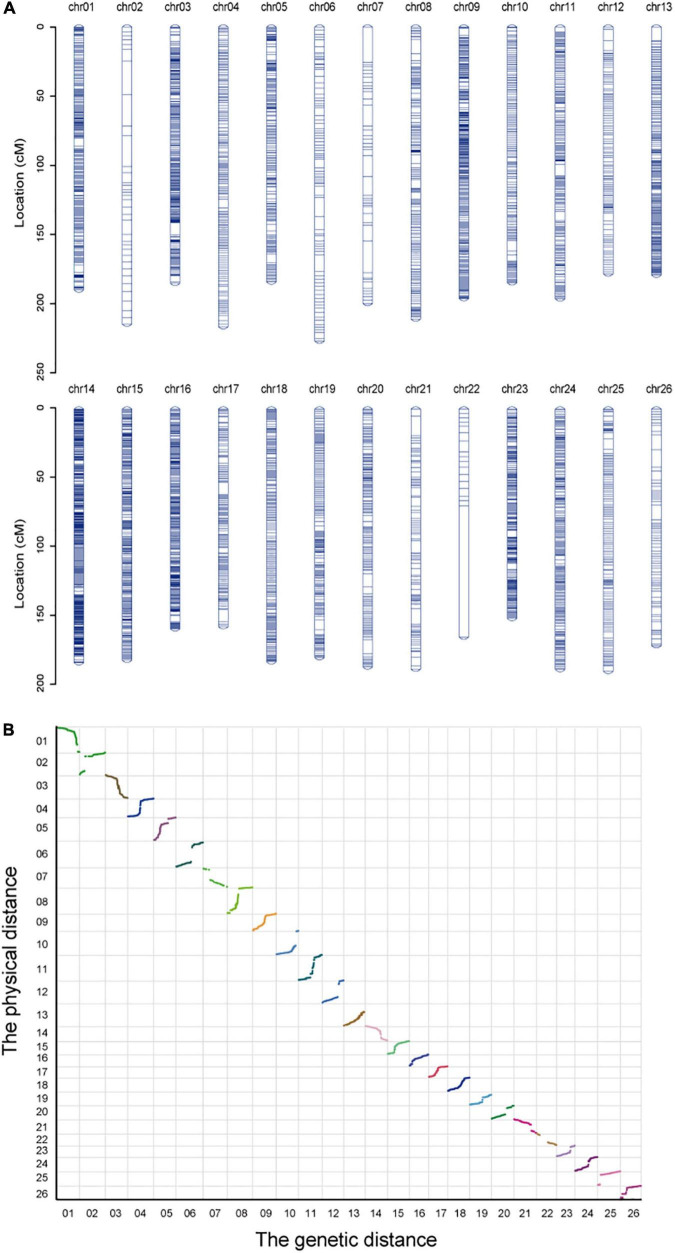
Detailed information about the genetic map. **(A)** The distribution of SLAF-SNP markers in the genetic map. **(B)** Collinearity analysis of markers between the physical map and genetic map.

A genetic map of 24,425 SNP markers and 4850.47 cM total distance over 26 chromosomes was built, having an average marker interval of 0.20 cM, wherein the A subgenome (A_*t*_) contained 14,220 SNP markers and 2564.93 cM and the D subgenome (D_*t*_) contained 10,205 SNP markers and 2285.54 cM (Figures 2A,B). The longest chromosome was chr06, which harbored 313 SNP markers with a length of 227.05 cM; conversely, the shortest was chr23 with 642 markers and a length of 151.39 cM. Using the Spearman coefficient for estimating the correlation with the physical mapping, except for chr08, the absolute Spearman values were all greater than 0.9 for all other chromosomes (Table 2).

**TABLE 2:** Detailed information on the linkage map for the CCRI70 population.

Chr	SNP marker number	Genetic distance (cM)	Avarage distance (cM)	Number of gap (>5 cM)	Spearman
chr01(A01)	966	190.12	0.20	2	0.99809
chr02(A02)	63	214.75	3.46	16	−0.98800
chr03(A03)	1781	185.26	0.10	1	0.99384
chr04(A04)	521	216.65	0.42	1	−0.99864
chr05(A05)	645	184.35	0.29	1	−0.99435
chr06(A06)	313	227.05	0.73	7	−0.98693
chr07(A07)	86	199.88	2.35	8	−0.98696
chr08(A08)	1576	211.13	0.13	4	−0.54889
chr09(A09)	2580	196.32	0.08	1	−0.99484
chr10(A10)	615	184.90	0.30	1	−0.97394
chr11(A11)	667	196.57	0.30	2	−0.99725
chr12(A12)	353	178.56	0.51	3	−0.99735
chr13(A13)	4054	179.39	0.04	1	−0.97012
chr14(D02)	2284	183.58	0.08	0	0.99700
chr15(D01)	1169	181.76	0.16	0	−0.99090
chr16(D07)	1106	159.11	0.14	0	−0.99883
chr17(D03)	603	157.45	0.26	2	−0.98077
chr18(D13)	587	182.94	0.31	0	−0.99828
chr19(D05)	1007	179.89	0.18	1	−0.99764
chr20(D10)	615	186.67	0.30	3	−0.99866
chr21(D11)	189	188.20	1.00	7	−0.99466
chr22(D04)	31	165.02	5.50	7	−0.93831
chr23(D09)	642	151.39	0.24	0	−0.99751
chr24(D08)	1387	188.51	0.14	0	−0.96497
chr25(D06)	361	189.79	0.53	1	−0.99851
chr26(D12)	224	171.22	0.77	6	−0.99459
Total	24425	4850.46	0.20	75	

### Quantitative Trait Loci and Quantitative Trait Loci Cluster Identification

Based on the high-density linkage map and phenotype data for the nine environments, 289 QTLs for fiber quality traits were detected on the whole genome. Specifically, 36 of them were stable QTLs, of which 15 were located on A_*t*_ and another 21 were detected on D_*t*_. The stable QTLs were mainly distributed on chromosomes 6, 10, 14, 16, 18, 24, and 25 (Supplementary Tables 3–5). Concerning the sign of QTL’s additive effect, a positive additive effect meant the high-quality related alleles originated from sGK-156, whereas negative meant the alleles were from 901-001.

**FIGURE 3:**
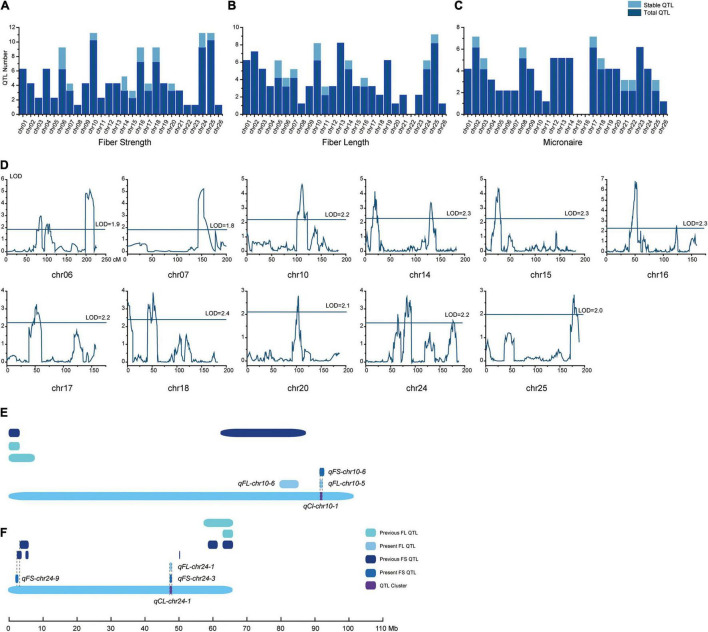
Detailed information about the QTLs. Total QTLs and stable QTLs of **(A)** fiber strength, **(B)** fiber length, and **(C)** micronaire distribution on the 26 chromosomes. **(D)** Fiber strength stable QTLs distribution on the 11 chromosomes. In **(E)** chr10 and **(F)** chr24, the FS and FL stable QTLs distribution in present and previous works.

There were 108 QTLs detected for FS, 17 of them (A_*t*_ was five and D_*t*_ was 12) were stable QTLs and mainly distributed on chromosomes 6, 14, 16, 18, and 24 (Figures 3A,D). Twelve of the 17 QTLs had negative additive effects, where the sGK156 alleles reduced the FS. In at least five environments, *qFS-chr18-1*, *qFS-chr16-4*, and *qFS-chr10-6* could be detected with negative additive effects.

Ninety-six QTLs were detected for FL, of which 11 (A_*t*_ had seven and D_*t*_ had four) were stable QTLs and mainly distributed on chromosome 5, 6, 10, 14, 24, and 25 (Figure 3B). Eight of the 11 QTLs had negative effects, where sGK156 alleles decreased the FL. *qFL-chr05-2*, *qFL-chr10-6*, and *qFL-chr24-1* could be detected in at least four environments with positive additive effects. Likewise, *qFL-chr05-3*, *qFL-chr10-5*, and *qFL-chr25-2* were detectable in four environments, albeit with negative additive effects.

In addition, 85 QTLs were identified for FM, among which eight (A_*t*_ had three and D_*t*_ had five) were stable QTLs (Figure 3C) and distributed on chromosome 2, 3, 8, 17, 18, 21, 22, and 25. Six of the eight QTLs had positive additive effects, whereby sGK156 increased the FM. In six environments, *qFM-chr03-2* could be detected with negative additive effects, whereas *qFM-chr08-3* and *qFM-chr22-1* were detectable in four environments with positive additive effects.

According to the overlapping of confidence intervals of stable QTLs for the different traits, seven QTL clusters (*qCl-chr07-1*, *qCl-chr10-1*, *qCl-chr14-1*, *qCl-chr16-1*, *qCl-chr17-1*, *qCl-chr24-1, qCl-chr25-1*) were identified on seven chromosomes (7, 10, 14, 16, 17, 24 and 25) (Supplementary Table 6), of which two were positioned on A_*t*_ and another five on D_*t*_. In five QTL clusters (*qCl-chr07-1*, *qCl-chr10-1*, *qCl-chr14-1*, *qCl-chr16-1, qCl-chr24-1*), the QTLs for FS and FL traits shared the same direction of additive effects. Besides, additive effects from *qCl-chr17-1* were in opposing directions for FS and FM, and likewise for *qCl-chr25-1* with respect to the FL and FM traits. These results suggested that the clusters could improve FS and FL simultaneously yet decrease the FM.

Comparing these stable QTLs with earlier published work for CCRI70 (Zou et al., 2018) and other research, 18 stable QTLs in the current study were novel and another 18 were either the same or shared overlapping physical confidence intervals with some in previous studies. Of these, 11 QTLs were matched those in the cotton QTL database, one was the same as found in the other CCRI70 study, two more partially overlapped with those reported in Zhang et al. (2020), and four more shared some overlap with previous GWAS results (Supplementary Table 7 and Figures 3E,F).

### Transcriptome Sequencing Analysis and Differentially Expressed Genes Identification

**FIGURE 4:**
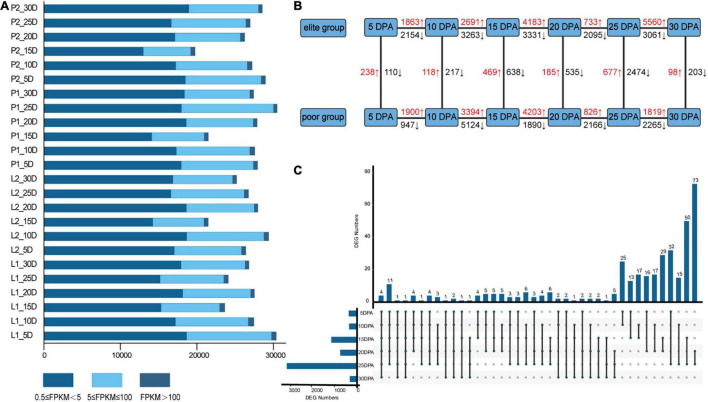
RNA-seq analysis statistics. **(A)** Statistics for transcript levels of each sample at each development stage, the numbers of expressed genes were divided by 0.5 < FPKM < 5, 5 < FPKM < 100, and FPKM > 100; **(B)** vertical and horizontal comparisons between different groups (elite group relative to the poor group) and stages; **(C)** the upset diagram showed the same and different DEGs identified at six developmental stages.

To reveal the patterns of gene expression during fiber developmental stages, transcriptome sequencing was conducted using fibers sampled at 5, 10, 15, 20, 25, and 30 DPA (Supplementary Table 8). As a result, 2,760.21 million clean reads were obtained. The raw data have been submitted to the National Genomics Data Center (accession numbers CRA002982 and CRA004731). The average number of clean reads, GC content, and Q30 value were, respectively, 38.34 million, 44.92%, and 94.93% for each sample, implying that the quality of the RNA-Seq data was reliable. After filtering out four low-correlation samples, the FPKM values were calculated for the biological replicates, and their percentages in the categories of 0.5 ≤ FPKM < 5, 5 ≤ FPKM ≤ 100, and FPKM > 100, respectively were 23.50, 12.24, and 0.78% on average (Figure 4A). Overall, 3,44,844 genetic variants were obtained on the 26 chromosomes referring to TM-1 when using the SNPEff program. As a result, 1,32,691; 1,85,414; 92,620; and 63,865 variants were detected in the 5’ UTR, 3’ UTR, exon and intron regions, respectively, where 52,097 were missense variants (Supplementary Table 9). Furthermore, *via* vertical comparisons, 238/110, 118/217, 469/638, 185/535, 677/2474, and 98/203 up-/downregulated DEGs relative to the inferior group for fiber traits were identified (Figure 4B). Meanwhile, in the horizontal comparisons, 24,266 unique DEGs were distinguished between different developmental stages, with 24,941 unique DEGs obtained overall. At the six developmental time points, four genes (*GH_A01G0307*, *GH_D02G0703*, *GH_D03G0833*, and *GH_D05G1628*) were significantly differentially expressed between two groups among all six stages. Meanwhile, 13 and 16 genes were expressed differently in five and four stages, respectively (Figure 4C). Detailed information for the FPKM of DEGs at different developmental stages, including their log_2_FoldChangeand FDR values, whether they were up- or downregulated, and also their respective annotations can be found in Supplementary Table 10.

### Weighted Gene Co-expression Network Analysis and Hub Genes Identification

Weighted gene co-expression network analysis was performed using 5,378 DEGs identified *via* the vertical comparisons. The topology overlap matrix was built with the hierarchical clustering method, and the dynamic tree cut modules, in which the modules shared high correlation (Supplementary Figure 1A). After merging the analogous expression patterns, 11 modules were finally identified (Supplementary Figure 1B). Among these, two modules (blue and brown) were specifically associated with high-quality groups at 5 and 25 DPA, when the fiber was respectively in the elongation and secondary wall biosynthesis stages. The pink module was specifically associated with low-quality groups at 30 DPA.

In the WGCNA, KME is a value that describes the eigengene connectivity. In this study, 22 hub genes were obtained with the KME values >0.94 (Supplementary Table 11). In the brown module, which was strongly associated with the high-quality group during fiber elongation, the hub genes were annotated as a disproportionating enzyme, CP12 domain-containing protein 3, cell elongation protein/DWARF1/DIMINUTO (DIM), 2-cysteine peroxiredoxin B, glyoxalase 2-4, nuclear-encoded CLP protease P7, and glucose-6-phosphate/phosphate translocator-related. *GH_D13G0256* was annotated as *DRF1* in *Arabidopsis thaliana*. It is involved in brassinosteroid biosynthesis, converting 24-methylenecholesterol to campesterol (Du and Poovaiah, 2005). Accordingly, *DRF1* loss-of-function mutants showed typical BR-deficient phenotypes, whereas *DWF1* overexpression enhanced growth and development in *A. thaliana* (Youn et al., 2018). In upland cotton, brassinosteroid was reported to play a significant role in fiber elongation as well (Yang et al., 2014; Tian and Zhang, 2021). In the blue module, the hub genes strongly associated with the high-quality group during secondary wall biosynthesis were annotated as B12D protein, sucrose-6F-phosphate phosphohydrolase family protein, calmodulin-domain protein kinase 9, RNA polymerase Rpb6, shaggy-like protein kinase 41, FK506-binding-like protein, staurosporin, and temperature-sensitive 3-like b and small ubiquitin-like modifier 1. Both *GH_A07G0466* and *GH_D07G0468* were identified as B12D proteins and separately distributed on At and Dt subgenomes. Similarly, *GH_A12G0434* and *GH_D12G0446* were annotated as shaggy-like protein kinase 41 and distributed on chromosomes A12 and D12, respectively.

### Identification of Promising Gene and Their Annotation

From the 36 stable QTLs, 1,476 promising genes were distinguished from 29 QTLs, whereas no genes were identified from the three QTLs (i.e., *qFS-chr07-2*, *qFL-chr05-3*, and *qFM-chr25-1*), with the narrow confidence intervals, and another four QTLs (*qFS-chr14-3*, *qFL-chr07-3*, *qFL-chr25-2*, and *qFM-chr08-3*) more than 20 MB in size on the physical map. Among those 1,476 unique promising genes, 1,005 of them were for the FS trait, 412 were for the FL trait, and 241 were for the FM trait (Supplementary Table 12). Among the stable QTLs, *qFS-chr17-2* harbored the most promising genes, 237, and six QTLs (*qFM-chr02-1*, *qFM-chr22-1*, *qFS-chr25-10*, *qFS-chr16-6*, *qFS-chr06-3*, and *qFL-chr24-1*) contained no more than 20 such genes. The *qFS-chr18-1* and *qFL-chr24-1*, which could be detected in most environments, contained 77 and 20 promising genes, respectively. Among the seven QTL clusters where 182 promising genes were identified, *qCl-chr10-1* had the most genes, with 54, whereas two clusters (*qCl-chr17-1* and *qCl-chr24-1*) had less than 20 each (Supplementary Figure 2).

The 1,476 promising genes were annotated with 4,109 GO terms. To the categories of biological process, cellular component, and molecular function belonged 2,602; 525; and 982 GO terms, respectively. In the three categories, 157, 82, and 87 genes were respectively enriched in terms of “regulation of transcription, “DNA-templated”, “cell wall”, and “sequence-specific DNA binding” (Supplementary Table 13).

Of the 1,476 promising genes, 834 were expressed (FPKM > 0.5), of which 246 were highly expressed (i.e., FPKM > 10). Comparing these promising genes with DEGs found revealed that 473 were expressed differentially during the process of fiber development, consisting of 320, 120, and 83 DEGs for FS, FL, and FM, respectively (Supplementary Table 9). Applying the KAAS for their pathway enrichment analysis, 473 differentially expressed promising genes were enriched in 130 pathways, these mainly for glycolysis/gluconeogenesis, protein processing in endoplasmic reticulum, amino sugar and nucleotide sugar metabolism, endocytosis, cysteine and methionine metabolism, pyruvate metabolism, phenylpropanoid biosynthesis, and plant-pathogen interaction pathway (Supplementary Table 13).

## Discussion

### Genetic Map Construction

When deriving a genetic map, it has often been constructed with restriction fragment length polymorphism (RFLP) markers, SSR markers, and SNP markers with gene chip and sequencing technologies (Ulloa et al., 2002, 2005; Rong et al., 2004; Chen et al., 2009; Sun et al., 2012; Wang P. et al., 2012; Zhang et al., 2012, 2016; Zhou et al., 2017; Zhang et al., 2020; Hulse-Kemp et al., 2015; Shi et al., 2015; Wang H. et al., 2015; Wang Y. et al., 2015; Yuan et al., 2015; Zhang Z. et al., 2015; Li et al., 2016; Liu et al., 2017; Qi et al., 2017; Chandnani et al., 2018; Diouf et al., 2018; Tan et al., 2018; Zou et al., 2018; Shi et al., 2020; Wang et al., 2021). The SNP marker development from next generation sequencing technology and genotyping with a reference genome provides a high-density molecular marker and unprecedented accuracy. In this study, a high-density genetic map containing 24,425 SNP markers and spanning a genetic distance of 4850.47 cM was constructed, wherein the high-density markers almost covered the entire genome of upland cotton. There were 75 just gaps (>5 cM) on the 26 chromosomes, and 38 of them were on chr02, chr07, chr21, and chr22, these being the chromosomes with the four fewest markers. In future work, we will consider increasing the marker density of these four chromosomes. Nonetheless, this genetic map provides a valuable new tool for studying QTLs and potential candidate gene identification and also informing cotton molecular breeding programs of upland cotton.

### Important Stable Quantitative Trait Loci Provided a Reference for Marker-Assisted Selection

Fiber quality traits are quantitative plant traits that are sensitive to the environment and other factors; so detecting QTLs in multiple environments was necessary to be done here, especially as it could improve accuracy. Some QTLs could be detected only in specific environments, whereas others that may be detected in multiple environments and across generations are considered as stable QTLs. In our study, the planting locations’ selection in experimental design took into account both geographic and temporal replicates. For example, Anyang and Linqing are located in the Yellow River basin while Alaer, Kuerle, and Shihezi are in the Xinjiang Autonomous Region. Meanwhile, Alaer, Anyang, and Linqing had been arranged for 2 years. It was our goal to detect the stable QTLs of upland cotton across multiple environments. Notably, of the36 stable QTLs, *qFL-chr11-2* and *qFM-chr02-1* were specifically detected in the Yellow River basin, whereas *qFM-chr17-2* was only identified in the Xinjiang Autonomous Region, and hence it might be environment-specific QTLs. Meanwhile, the fact that *qFS-chr18-1*, *qFL-chr24-1*, and *qFM-chr03-2* were detected in at least six environments is strong evidence suggesting that genetic factors may play a dominant role in fiber quality traits.

**FIGURE 5:**
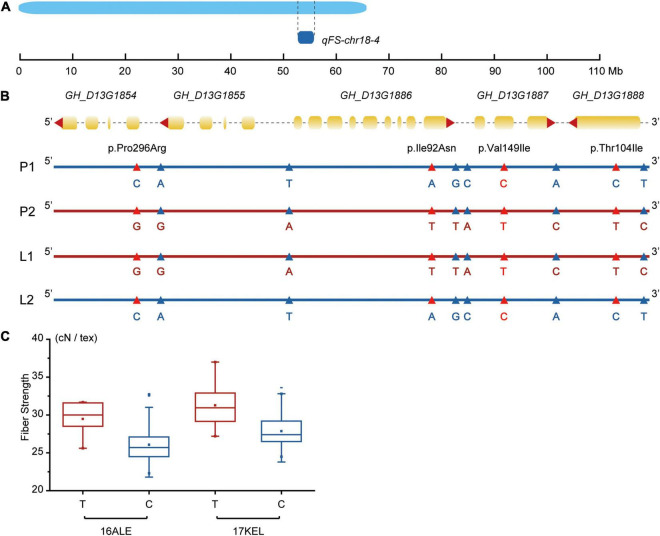
Detailed genetic variants distribution in qFS-18-4. **(A)** The distribution of qFS-18-4 in chr18. **(B)** Ten different SNPs between high- and low-group distributed in the QTL. The SNP leading to missense variants were marked as red triangle, whereas the variants located on UTR were marked as a blue triangle. Marker GH_D13G1855 was marked in red. **(C)** In marker D13_56413025, the phenotypes of the materials having T and C showed significant differences (*p* < 0.05) in 16ALE and 17KEL.

Among the 36 stable QTLs found in this study, 18 had been identified before (eight for FS, six for FL, and four for FM), but the other 18 were novel (nine for FS, five for FL, and four for FM). Together, they provide a sound basis for further functional characterization and upland cotton marker-assisted breeding programs. Our work differs from previous research, in that the CCRI70 RIL population is a breeding population developed from two upland cotton cultivars, which themselves are breeding material rather than germplasm material. Moreover, CCRI70 (F_1_) was the first nationally approved hybrid cotton capable of a high yield of superior fiber quality. The goal of this study was to detect QTLs and identify promising genes related to key fiber quality traits, which could be used to reveal the parental source of fiber quality related alleles. Compared with previous QTL studies of fiber quality, few fiber quality related QTLs, especially FS-related QTLs, were identified on chr18 (D13) (Diouf et al., 2018; Gu et al., 2020; Wang et al., 2020; Zhang et al., 2020). However, we detected two stable QTLs related with FS and one related with FM on that chromosome, and the *qFS-chr18-1* was detected in eight of the nine testing environments. Next, focusing on the novel QTLs, we combined DNA sequencing and RNA-Seq data to explore the molecular mechanisms of fiber quality traits and genetic variants’ distribution in the QTLs among the high- and low-fiber quality group. In *qFS-chr18-4* (Figure 5A), 901-001 increased the FS, for which 19 SNPs were identified from DNA sequencing data and 10 were detected from RNA-Seq data, which performed differently between the two groups. The 10 SNPs were located in the mRNA of five promising genes (Figure 5B); four led to missense variants and six were detectable on the 5’/3’ UTR. Besides, the high- and low-fiber quality groups we studied had some differences and also same genotypes with TM-1 (Hu et al., 2019), which suggests the variants could increase FS. By scanning the SNPs in the natural population (Zhang et al., 2020) and reanalyzing the genotypic data using the newest reference genome (Hu et al., 2019) for marker D13_56413025, the phenotypes of the materials having the same and different allele with 901-001 showed significant differences at 16ALE and 17KEL environments, with *p*-values < 0.05. In 16ALE, the FS phenotypes of materials with T spanned 25.60-31.70 cN/tex with an average of 29.48 cN/tex, while the phenotypes with C were 22.70–32.80 cN/tex with an average of 26.08 cN/tex. Meanwhile, in 17KEL, the range of the holding T allele was 27.20–37.00 cN/tex, averaging 31.28 cN/tex, whereas the range of C allele was 24.50–33.60 cN/tex with an average of 27.86 cN/tex. The FS increased from 3.40 to 3.41 cN/tex (Figure 5C). The marker D13_56413025 also led to missense variant in the highly expressed promising gene *GH_D13G1887* (FPKM > 10), making it worthy of further study and providing information to better understand the underlying genetic mechanisms, as well as a reference for improving the upland cotton fiber quality.

### Significant Promising Genes Reveal Key Pathways Involved in Fiber Strength

The promising genes identification was confirmed by the CI of stable QTLs, whose dynamics of expression are of significance for functional characterization. We identified 24,941 unique DEGs through the comparative transcriptome analysis (Supplementary Table 8) and 1,476 promising genes were detected in stable QTLs (Supplementary Table 9). A total of 473 of them expressed differentially during fiber development and were enriched in 130 pathways according to their KEGG analysis (Supplementary Table 12). These promising genes were all highly expressed DEGs (FPKM > 10) and enriched in critical pathways, as expected of significant promising genes. It is the primary and secondary cell wall deposition that determines the final FS trait (Pang et al., 2010). In our study, 10 significant promising genes enriched in the biological processes of pectin synthesis, phenylpropanoid biosynthesis, plant hormone signaling pathways, and transcription factors had an impact on FS and might regulate the process of fiber development in upland cotton (Supplementary Figure 3).

Three promising genes (*GH_A06G0729, GH_D02G0231*, *GH_D11G1746*) were enriched in the amino sugar and nucleotide sugar metabolism pathway. Among them, *GH_D02G0231* was located in *qFS-chr14-1* and identified as UDP-D-glucuronate 4-epimerase 6 (GAE6). UDP-d-galacturonate is necessary for pectin’s synthesis, in that the latter is synthesized from UDP-d-glucuronate by GAE6 (Usadel et al., 2004). Furthermore, *GAE6* was found involved in both cotton fiber and *Arabidopsis* root hair growth (Pang et al., 2010). Another five promising genes (*GH_A06G0665*, *GH_D10G2039*, *GH_D13G2625*, *GH_D13G1855*, and *GH_D13G2599*) were enriched in the phenylpropanoid biosynthesis pathway and annotated as aldehyde dehydrogenase 2C4, CINNAMATE 4-HYDROXYLASE (C4H), O-METHYLTRANSFERASE 1 (OMT1), and AMP-dependent synthetase and ligase family protein. Interestingly, *GH_D10G2039*, *GH_D13G2625*, and *GH_D13G1855* each exhibited high expression levels during fiber developmental stages and were identified in *qFS-chr20-1*, *qFS-chr18-1*, and *qFS-chr18-4*, respectively. All currently known building blocks of the lignin polymer are produced by, or derived from the general phenylpropanoid pathway (Barros et al., 2016; Zhou et al., 2017; Vanholme et al., 2019), which suggests that *OMT1* and *C4H* play key functions in the pathway and influence FS (Guo et al., 2001; Schilmiller et al., 2009; Sadeghi et al., 2013; Vanholme et al., 2013; Sundin et al., 2014; Ho-Yue-Kuang et al., 2016). In addition, one missense variant and one upstream gene variant were detected here in *GH_D13G2625* and *GH_D13G1855* between the high- and low-quality groups. Six promising genes (*GH_A06G0641*, *GH_D07G1483*, *GH_D07G1512*, *GH_D08G0287*, *GH_D13G2571*, *GH_D13G2572*) were enriched in the plant hormone signal transduction pathway. *GH_D07G1483* was enriched into auxin-signaling pathway as AUXIN RESPONSE FACTOR 18 (ARF18) and located in *qCl-chr16-1*, a genome region that influences both the FS and FL of upland cotton. Through phylogenetic analysis, ARF18 was classified into the class containing ARF1, ARF2, ARF9, and ARF11 (Okushima et al., 2005), all of which function as transcription repressors (Ulmasov et al., 1997). In *Brassica napus* L, ARF18 regulates cell growth in the silique wall and determines the seed weight (Liu J. et al., 2015). *GH_D13G2571* was identified in *qFS-chr18-1* and annotated as a transcription factor (TF) ETHYLENE INSENSITIVE 3 (EIN3), which is considered the key transcriptional regulator of the ethylene response in plants (Dolgikh et al., 2019) and accordingly has the most prominent role in ethylene signaling (Alonso et al., 2003; Binder et al., 2007; Binder, 2020). Meanwhile, *GH_D07G1512*, located in both *qFL-chr16-3* and *qFS-chr16-4*, was annotated as JASMONATE RESISTANT1 (JAR1), which could activate JA signaling (Meesters et al., 2014). *GH_D13G2571* and *GH_D07G1512* were enriched in the ethylene signaling and jasmonic acid (JA) signaling pathways, respectively. *EIN3* could regulate *ETHYLENE RESPONSE FACTOR 1* (*ERF1*), and *GbERF1-like* reportedly activates the JA/ET signaling pathway and lignin synthesis (Guo et al., 2016). In addition, they reached their peak expression levels at 25 DPA, when the fiber cell was in the secondary wall biosynthesis stage, thus suggesting that they regulate the ethylene and jasmonic acid pathways and thus affect FS. Further, there were three missense variants and two synonymous variants identified in *GH_D13G2571* between the high- and low-quality groups, a finding that merits further investigation. Besides, another three transcription factors were identified, which might regulate fiber cell development and affect FS. *GH_D06G0054* was annotated here as transcription factor bZIP44. It has been reported that *AtbZIP44* TF could positively regulate *AtMAN7* expression and influence not only the loosening of the cell wall in the micropylar endosperm upon germination but could also be involved in the elongation of radicle cells (Iglesias-Fernández et al., 2013). *GH_A06G0641* was annotated as DELLA family member GIBBERELLIC ACID INSENSITIVE (GAI), whose mutants were shown to reduce GA responses. *GAI* is involved in regulating stem elongation *via* the GA signaling pathway (Peng et al., 1997; Tan et al., 2019), which also plays a crucial role in trichome development (Wang Z. et al., 2019). *GH_D01G2072* was identified as TEOSINTE BRANCHED1, CYCLOIDEA, PCF8 (TCP8) belonging to the TCP family class I group (Cubas et al., 2001), for which gene redundancy is a common feature. The TCP family class I group was shown to be involved in regulating leaf development (Aguilar Martinez and Sinha, 2013). Both *TCP8* and *TCP15*, other members of this member of the family class I group, could regulate filament elongation (Gastaldi et al., 2020). Moreover, *GhTCP14*, a class I member in upland cotton, was also identified as producing higher transcripts during fiber initiation and elongation in *G. hirsutum*, which enhanced the abundance of trichomes on the stem, inflorescence, and root parts (Wang et al., 2013; Wang Z. et al., 2019). To sum up, within the stable QTLs, it was the 10 significant promising genes related to pectin synthesis, phenylpropanoid biosynthesis, plant hormone signaling pathways as well as some transcription factors that played vital roles in determining the FS trait of upland cotton.

## Conclusion

To investigate the parental source of high-quality related alleles, we constructed a genetic map of the hybrid CCRI70 RIL population by using SLAF-seq, this containing 24,425 SNP markers spanning a distance of 4850.47 cM. To detect stable QTL across multiple environments, we evaluated three key fiber quality traits in nine environments at six locations in 2015 and 2016. This revealed 289 QTLs, 36 of these were stable QTLs (17 for FS, 11 for FL, and eight for FM), of which 15 were located on A_*t*_ and another 21 detected on D_*t*_. Comparing the stable QTLs with those in previous studies, 18 were already known (eight for FS, six for FL, and four for FM) yet the other 18 were novel (nine for FS, five for FL, and four for FM); they provide a robust basis for understanding the distribution and the sources of high fiber quality-related alleles in cotton plants. Notably, *qFS-chr18-4* and marker D13_56413025 could serve as important references for upland cotton maker-assisted selection breeding. Transcriptome sequencing for 72 libraries of the two parents and two RILs during fiber development with three biological repeats was performed, this aiming to understand the potential candidate genes expression patterns and DEGs (differentially expressed genes) in superior and inferior fiber quality groups during the process of fiber development. Based on this transcriptome analysis, 24,941 unique DEGs were identified *via* vertical and horizontal comparisons, from which 473 of the 1,476 promising genes were identified as DEGs during fiber development. Among 320 differentially expressed promising genes for FS, 10 significant promising genes with high expression levels were enriched in the pathways of pectin synthesis, phenylpropanoid biosynthesis, and plant hormone signaling; these, as well as several others, annotated as transcription factors may jointly impact FS and regulate fiber development. Altogether, our results provide a fresh basis for improving cotton fiber quality *via* marker-assisted breeding that could benefit the textile industry.

## Data Availability Statement

The datasets presented in this study can be found in online repositories. The names of the repository/repositories and accession number(s) can be found below: https://ngdc.cncb.ac.cn/, (CRA002982 and CRA00473).

## Author Contributions

XJ: conceptualization, formal analysis, validation, software, and writing—original draft. JG: resources. JZ, HC, ZK and JP: investigation. ZZ: software and methodology. YS: methodology. JL, QG, and SZ: visualization. AL and WG: data curation. XD and SF: project administration. JC: formal analysis. TJ and RW: software. QC: investigation and writing—review and editing. SW: writing—review and editing. HS and YY: conceptualization, writing—review and editing, supervision, and funding acquisition. All authors contributed to the article and approved the submitted version.

## Conflict of Interest

The authors declare that the research was conducted in the absence of any commercial or financial relationships that could be construed as a potential conflict of interest.

## Publisher’s Note

All claims expressed in this article are solely those of the authors and do not necessarily represent those of their affiliated organizations, or those of the publisher, the editors and the reviewers. Any product that may be evaluated in this article, or claim that may be made by its manufacturer, is not guaranteed or endorsed by the publisher.

## Footnotes

1
http://www.cottonqtldb.org

2
https://cottonfgd.org/

3
http://www.genome.jp/kegg/kaas/

4
http://planttfdb.cbi.pku.edu.cn/

## Supplementary Material

Supplementary Figure 1 Weighted gene co-expression network analysis (WGCNA) of DEGs. **(A)** Hierarchical dendrogram showing co-expression modules identified by WGCNA. Each leaf in the tree represents one gene. The major tree was divided into 11 modules in total. **(B)** Module–sample relationships. With the correlation coefficient and the e-value shown in each square, each column represents a sample tissue and replication.

Supplementary Figure 2 Heatmap of expression in QTL clusters. **(A)** qCl-chr10-1, **(B)** qCl-chr14-1, **(C)** qCl-chr16-1, **(D)** qCl-chr17-1, and **(E)** qCl-chr24-1 distribution and heatmaps of potential candidate genes expression [log_2_(FPKM + 1)].

Supplementary Figure 3 Dynamics of 10 significant promising genes’ expression. Expression dynamics of *GH_D02G0231* **(A)**, *GH_D10G2039* **(B)**, *GH_D13G2625* **(C)**, *GH_D13G1855* **(D)**, *GH_D07G1483* **(E)**, *GH_D13G2571* **(F)**, *GH_D07G1512* **(G)**, *GH_D06G0054* **(H)**, *GH_A06G0641* **(I)**, and *GH_D01G2072* **(J)** in fiber developmental stages.
