# Supplementary figures and images for: Quantitative Trait Loci and Transcriptome Analysis Reveal Genetic Basis of Fiber Quality Traits in CCRI70 RIL Population of Gossypium hirsutum
Source: Front Plant Sci. 2021 Dec 16;12:753755. doi: 10.3389/fpls.2021.753755 (PMC8716697; doi:10.3389/fpls.2021.753755)

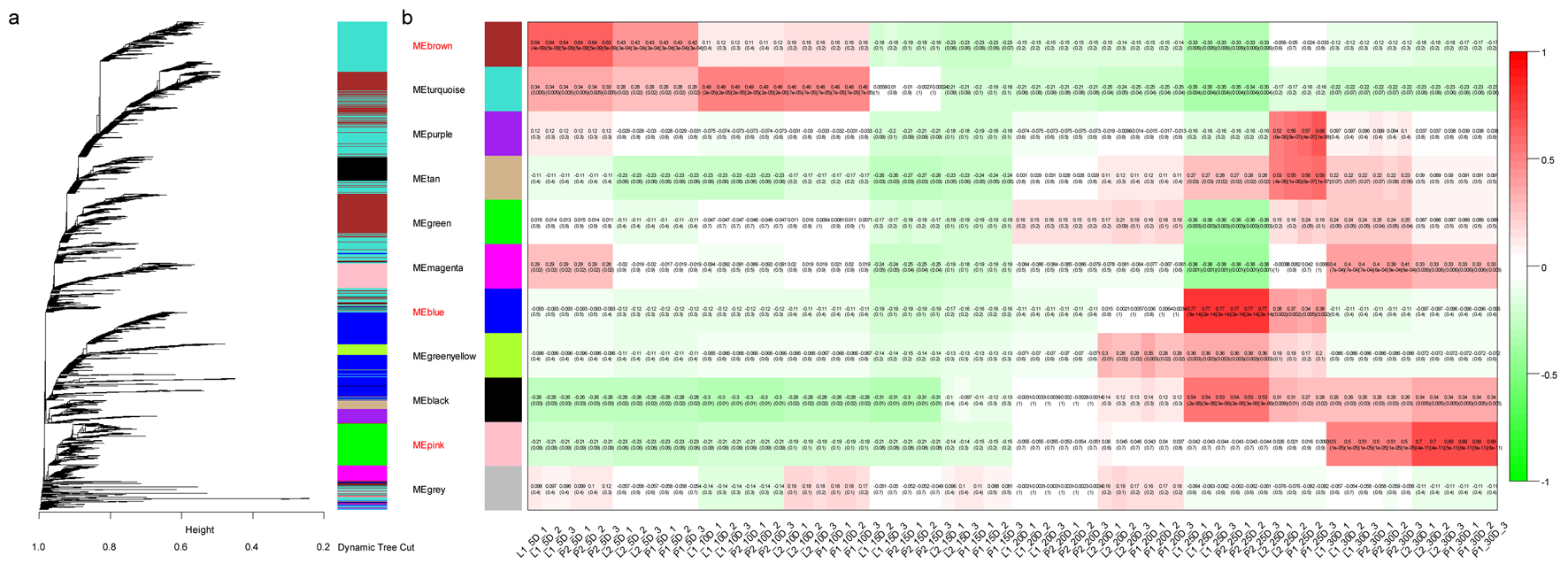

Supplement: Supplementary Figure 1 — Weighted gene co-expression network analysis (WGCNA) of DEGs. (A) Hierarchical dendrogram showing co-expression modules identified by WGCNA. Each leaf in the tree represents one gene. The major tree was divided into 11 modules in total. (B) Module–sample relationships. With the correlation coefficient and the e-value shown in each square, each column represents a sample tissue and replication. [file Image_1.TIF]

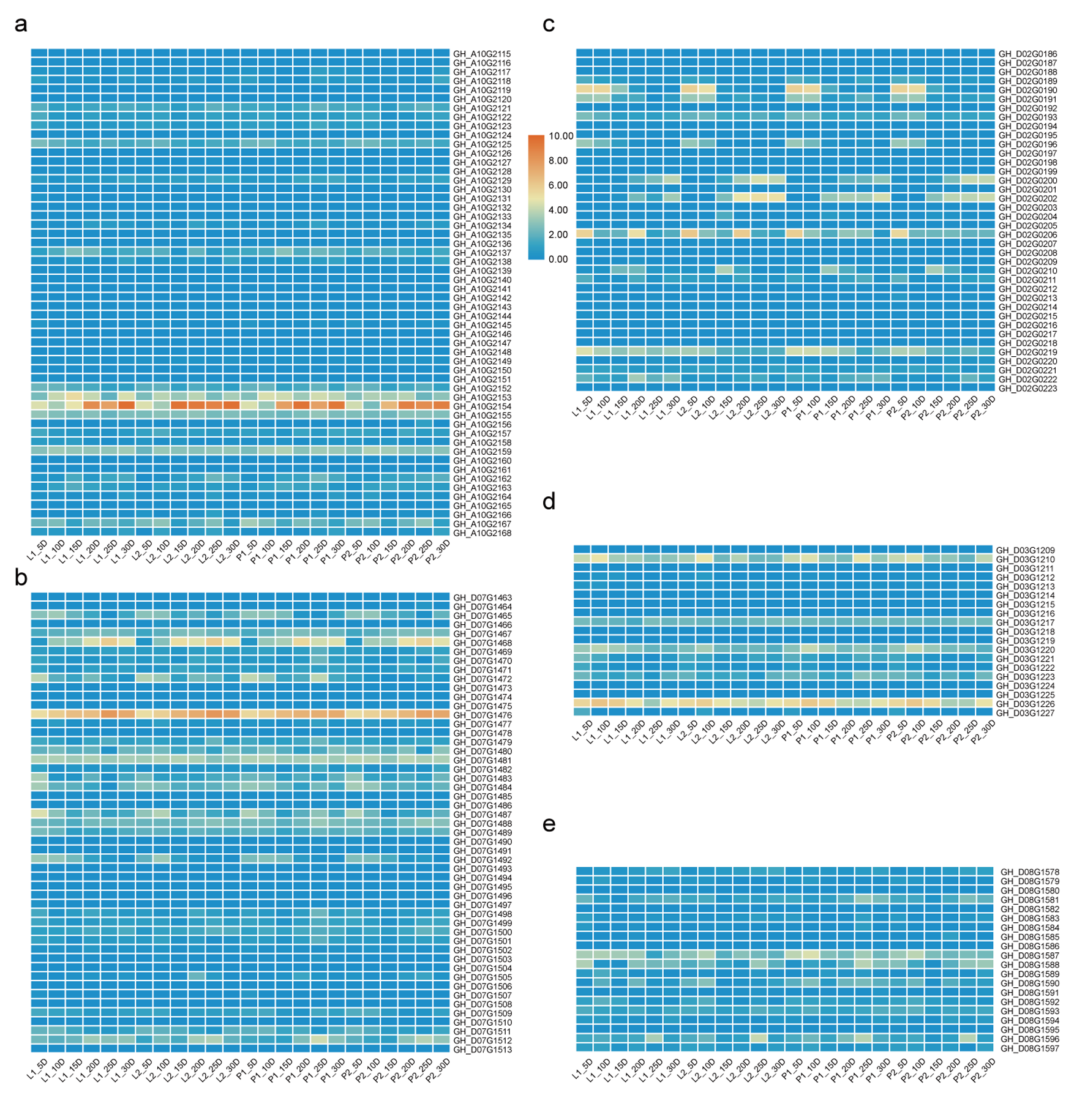

Supplement: Supplementary Figure 2 — Heatmap of expression in QTL clusters. (A) qCl-chr10-1, (B) qCl-chr14-1, (C) qCl-chr16-1, (D) qCl-chr17-1, and (E) qCl-chr24-1 distribution and heatmaps of potential candidate genes expression [log2(FPKM + 1)]. [file Image_2.TIF]

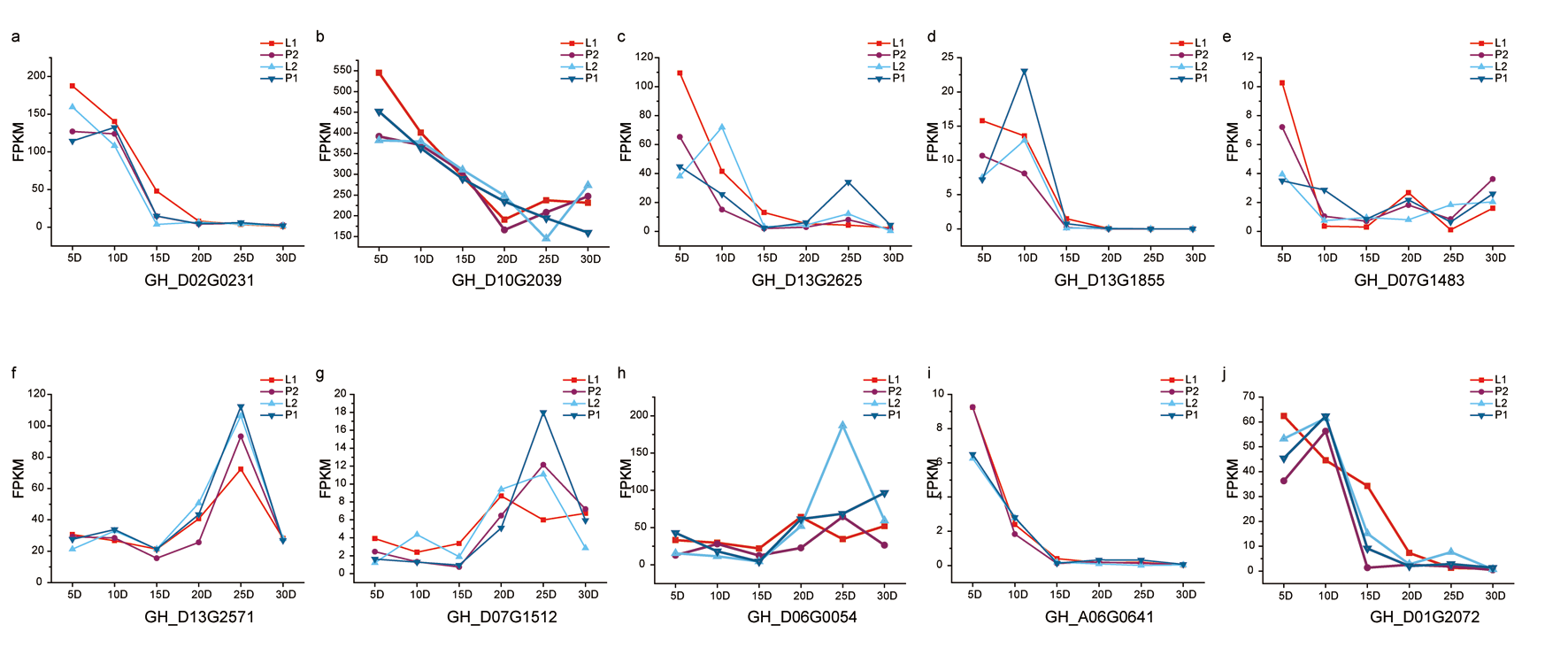

Supplement: Supplementary Figure 3 — Dynamics of 10 significant promising genes’ expression. Expression dynamics of GH_D02G0231 (A), GH_D10G2039 (B), GH_D13G2625 (C), GH_D13G1855 (D), GH_D07G1483 (E), GH_D13G2571 (F), GH_D07G1512 (G), GH_D06G0054 (H), GH_A06G0641 (I), and GH_D01G2072 (J) in fiber developmental stages. [file Image_3.TIF]
